# Shiga Toxins Induce Apoptosis and ER Stress in Human Retinal Pigment Epithelial Cells

**DOI:** 10.3390/toxins9100319

**Published:** 2017-10-13

**Authors:** Jun-Young Park, Yu-Jin Jeong, Sung-Kyun Park, Sung-Jin Yoon, Song Choi, Dae Gwin Jeong, Su Wol Chung, Byung Joo Lee, Jeong Hun Kim, Vernon L. Tesh, Moo-Seung Lee, Young-Jun Park

**Affiliations:** 1 Metabolic Regulation Research Center, Korea Research Institute of Bioscience and Biotechnology, 125 Gwahak-ro, Daejeon 34141, South Korea; pi1812@kribb.re.kr (J.-Y.P.); sjy04@kribb.re.kr (S.-J.Y.); ssong@kribb.re.kr (S.C.); 2 Infectious Disease Research Center, Korea Research Institute of Bioscience and Biotechnology, 125 Gwahak-ro, Daejeon 34141, South Korea; lfllying@kribb.re.kr (Y.-J.J.); skpark@kribb.re.kr (S.-K.P.); dgjeong@kribb.re.kr (D.G.J.); 3 Department of Microbial Pathogenesis and Immunology, Texas A&M University Health Science Center, Bryan, TX 77807, USA; tesh@medicine.tamhsc.edu; 4 Department of Biochemistry, College of Medicine, Konyang University, 158 Gwanjeo-ro, Daejeon 35365, South Korea; 5 Department of Biomolecular Science, KRIBB School of Bioscience, Korea University of Science and Technology (UST), 127 Gajeong-ro, Yuseong-gu, Daejeon 34113, South Korea; 6 School of Biological Sciences, College of Natural Sciences, University of Ulsan, 93 Daehak-ro, Ulsan 44610, South Korea; swchung@ulsan.ac.kr; 7 Fight Against Angiogenesis-Related Blindness Laboratory, Biomedical Research Institute, Seoul National University Hospital, Seoul 03080, South Korea; ozma805@empas.com (B.J.L.); steph25@snu.ac.kr (J.H.K.)

**Keywords:** Shiga toxins, Shiga toxin type 1 and 2, Shiga toxin-producing *Escherichia coli*, hemolytic uremic syndrome, signaling pathways, apoptosis, retinal pigment epithelial cells

## Abstract

Shiga toxins (Stxs) produced by Shiga toxin-producing bacteria *Shigella dysenteriae* serotype 1 and select serotypes of *Escherichia coli* are the most potent known virulence factors in the pathogenesis of hemorrhagic colitis progressing to potentially fatal systemic complications such as acute renal failure, blindness and neurological abnormalities. Although numerous studies have defined apoptotic responses to Shiga toxin type 1 (Stx1) or Shiga toxin type 2 (Stx2) in a variety of cell types, the potential significance of Stx-induced apoptosis of photoreceptor and pigmented cells of the eye following intoxication is unknown. We explored the use of immortalized human retinal pigment epithelial (RPE) cells as an in vitro model of Stx-induced retinal damage. To the best of our knowledge, this study is the first report that intoxication of RPE cells with Stxs activates both apoptotic cell death signaling and the endoplasmic reticulum (ER) stress response. Using live-cell imaging analysis, fluorescently labeled Stx1 or Stx2 were internalized and routed to the RPE cell endoplasmic reticulum. RPE cells were significantly sensitive to wild type Stxs by 72 h, while the cells survived challenge with enzymatically deficient mutant toxins (Stx1A^−^ or Stx2A^−^). Upon exposure to purified Stxs, RPE cells showed activation of a caspase-dependent apoptotic program involving a reduction of mitochondrial transmembrane potential (Δψ_m_), increased activation of ER stress sensors IRE1, PERK and ATF6, and overexpression CHOP and DR5. Finally, we demonstrated that treatment of RPE cells with Stxs resulted in the activation of c-Jun N-terminal kinase (JNK) and p38 mitogen-activated protein kinase (p38MAPK), suggesting that the ribotoxic stress response may be triggered. Collectively, these data support the involvement of Stx-induced apoptosis in ocular complications of intoxication. The evaluation of apoptotic responses to Stxs by cells isolated from multiple organs may reveal unique functional patterns of the cytotoxic actions of these toxins in the systemic complications that follow ingestion of toxin-producing bacteria.

## 1. Introduction

Shiga toxins (Stxs) are genetically, structurally, and functionally conserved protein exotoxins produced by several bacterial species, including *Shigella dysenteriae* serotype 1 and Stx-producing *Escherichia coli* (STEC). Following ingestion and adherence of STEC in the intestinal tract, patients may experience bloody diarrhea followed by a complicated and potentially fatal disease course that frequently includes microangiopathic hemolytic anemia, thrombocytopenia and acute renal failure, also known as hemolytic uremic syndrome (HUS), and neurological complications [1]. Stxs are critical virulence determinants in these systemic complications. The neutral glycolipid globotriaosylceramide (Gb3) serves as the toxin receptor on the surface of host cells, and sites of tissue damage often correlate with Gb3 expression [2,3,4,5]. Once Stxs are internalized following Gb3 receptor binding, they are trafficked in a retrograde manner into early endosomes, and then through the *trans*-Golgi network and Golgi apparatus to reach the endoplasmic reticulum (ER). Stxs cross the ER membrane to enter the cytosol [6,7,8]. All Stxs are ribosome-inactivating proteins and possess an AB5 configuration comprised of a monomeric enzymatic A-subunit in non-covalent association with a pentameric ring of identical B-subunit proteins [9,10]. Following retro-translocation across the ER membrane, a processed fragment of the toxin enzymatic A-subunit cleaves a single adenine residue from the 28S rRNA component of the ribosome, leading to host cellular protein synthesis inhibition and induction of apoptosis through ER stress signaling [11,12,13,14,15]. Despite the fact that STEC constitute a significant public health concern, the pathophysiology of systemic complications following ingestion of toxin-producing bacteria is not well understood. We and others have shown that the administration of purified Stxs to mice or baboons reproduced many of the pathophysiologic changes seen patients infected with the toxin-producing bacteria, including intestinal, renal and central nervous system (CNS) damage [16,17,18,19,20,21]. Histologic studies of HUS cases showed that Stxs target vascular endothelial cells for destruction, leading to blood vessel damage in the intestine, kidneys and CNS [22,23,24,25]. 

A strong association between neurological involvement and impairment of the visual system has been reported in pediatric HUS patients [26]. Recently, a 10-month-old pediatric patient infected with Shiga toxin-producing *Escherichia coli* O104 developed lethargy that necessitated admission to the intensive care unit. The patient presented with severe HUS with retinal and choroidal hemorrhages, as well as ischemic events due to thrombotic microangiopathic lesions. After three months, the infant neurologically had minor physical disabilities and no apparent cognitive disabilities and was discharged from the hospital with complete blindness and severe chronic renal failure [27]. Thus, physicians have become aware of ocular involvement in STEC-mediated HUS because of possible vision-endangering consequences. Retinal pigment epithelium (RPE) found at the base of the retina are just posterior to the photoreceptors, a specialized type of neuron in the retina. Photoreceptors are capable of converting light into signals for vision by stimulating neuronal impulse transmission [28]. Polarized RPE cells are essential for maintaining the proper visual function in the retinal physiology. However, despite recent clinical case reports in which patients present with ocular involvement, there are no precise mechanisms defined by which Stxs contribute to the injury of RPE cells that are closely associated with proper visual function. Thus, we determined whether Stx1- and Stx2-induced apoptosis with toxins induced the ribotoxic and ER stress response signaling using the ARPE-19 human retinal pigment epithelial cell line. In the present study, we first report that receptor Gb3-dependent Stx endocytosis activates the MAPK-mediated ribotoxic stress response and apoptotic and ER stress pathways, triggering caspase-3/7/8 cleavage as well as disrupting the mitochondrial membrane potential in the newly identified toxin-sensitive RPE cell line ARPE-19.

## 2. Results

### 2.1. ARPE-19 Cells Are Sensitive to the Cytotoxic Effects of Stx1 and Stx2

**Figure 1:**
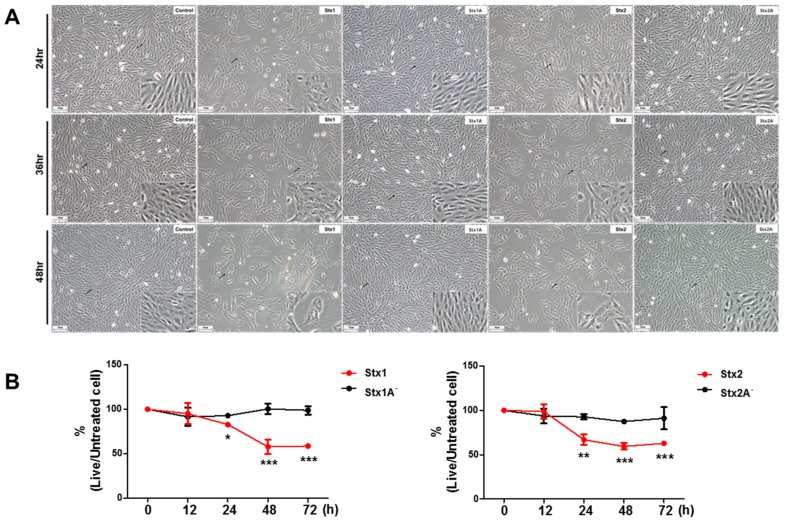
The effects of Shiga toxin treatment on cell death in ARPE-19 cells. (**A**) Stx1 or Stx2 treatment, but not Stx1A^−^ or Stx2A^−^ treatment, show dramatic changes in cell morphology. Images were collected from cells incubated with or without Stxs for 24, 36, and 48 h; (**B**) ARPE-19 cells were seeded in 96-well plates at a total cell density of approximately 1.0 × 10^4^ cells/well. Cells were incubated with Stx1 (100 ng/mL), Stx1A^−^ (100 ng/mL), Stx2 (10 ng/mL) or Stx2A^−^ (10 ng/mL) for the indicated time points. Cell viability was measured by colorimetric assay using the dye tetrazolium compound [3-(4,5-dimethylthiazol-2-yl)-5-(3-carboxymethoxyphenyl)-2-(4-sulfophenyl)-2*H*-tetrazolium] inert salt (MTS). Data are expressed as % viability compared to untreated control cells at each time point. Results shown are mean ± SEM from two independent experiments using triplicate samples. Asterisks denote statistical significance, Stx1 or Stx2 treatment vs. Stx1A− or Stx2A− treatment, * = *p* < 0.05; ** = *p* < 0.01; *** = *p* < 0.001.

Previous studies have indicated that Stxs induce cytotoxic effects in various cell types including monocytic, macrophage-like, and epithelial cell lines [11,29]. To establish the effect of Stxs on ARPE-19 cells, we first investigated the morphologic features of ARPE-19 cells when treated with Stx1 (100 ng/mL), Stx1A^−^ (100 ng/mL), Stx2 (10 ng/mL), or Stx2A^−^ (10 ng/mL). ARPE-19 cells presented the typical morphology under control conditions, while Stx1- and Stx2-treated cells exhibited dramatic morphological changes and cytopathic effects at the indicated incubation times. However, both Stx1A^−^ and Stx2A^−^ which lack enzymatic activity due to mutations in the A subunit catalytic residue of each toxin, showed similar features to control cells (Figure 1A). The cytotoxic effects of Stxs on ARPE-19 cells were assessed by cell viability measurements following the incubation of cells with Stx1 (100 ng/mL) and Stx2 (10 ng/mL) for 0–72 h. Cell viability rapidly decreased beginning 24 h after incubation with Stxs. In contrast, major changes in cell viability were not detected after 24 h of exposure of ARPE-19 cells to Stxs with mutations in the A subunit (Figure 1B). As shown in Figure S1, a dose- and time-dependent increase of cytotoxicity was observed for all Stxs (Stx1 and Stx2) at the range of concentrations from 1.0 to 400 ng/mL. CD_50_ values of ~100 ng/mL and ~10 ng/mL were estimated for Stx1 and Stx2, respectively. These results indicate that ARPE-19 cells are sensitive to Stx-induced cytotoxicity. This makes RPEs much less sensitive to Stxs compared to Vero or HeLa cells that have CD_50s_ in the pg/mL [15].

### 2.2. ARPE-19 Cells Express Membrane-Associated Stx-Receptor Gb3 Expression at the Cell Surface

**Figure 2:**
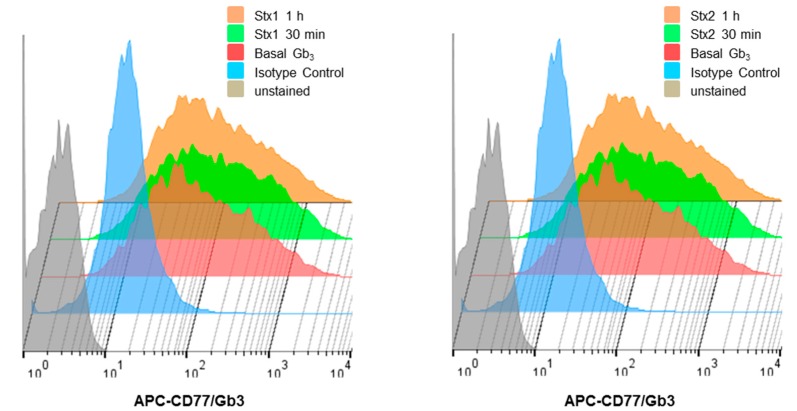
Analysis of Gb3 expression on ARPE-19 cells by flow cytometry. ARPE-19 cells were stained with Alexa Fluor 647-conjugated anti-Gb3/CD77 antibody or an isotype-matched antibody (mouse IgM-Alexa Fluor 647) to determine the background fluorescence at 4 °C for 30 min. or after 30 min or 1 h of stimulation with (left panel) Stx1 (100 ng/mL) or (right panel) Stx2 (10 ng/mL).

As reported previously, the binding of Stxs to cell surfaces requires specific glycolipids, with globotriaosylceramide (Gb3) being essential for toxin internalization and subsequent cytotoxicity [5]. Specific cell types in the kidney expressing high Gb3 levels are much more sensitive to the cytotoxic effects of Stxs compared to non-kidney cells with lower Gb3 expression (e.g., human brain endothelial cells) [30,31,32]. To correlate the cytotoxicity of Stxs with Gb3 expression, we examined Gb3 expression on the surface of ARPE-19 cells. Basal levels of Gb3 expression on the cell surface were measured by Fluorescence- Activated Cell Sorter (FACS) analysis using monoclonal anti-Gb3/CD77 antibodies (Figure 2). Cultured ARPE-19 cells showed expression of Gb3. Levels of Gb3 expression in ARPE-19 cells treated with Stxs at early time points were not significantly altered. In summary, the ARPE-19 cell line expressed Gb3 on the cell surface but did not show changes in Gb3 expression when treated with Stxs for up to 1 h. 

### 2.3. Intracellular Trafficking of Stxs to the ER in ARPE-19 Cells

**Figure 3:**
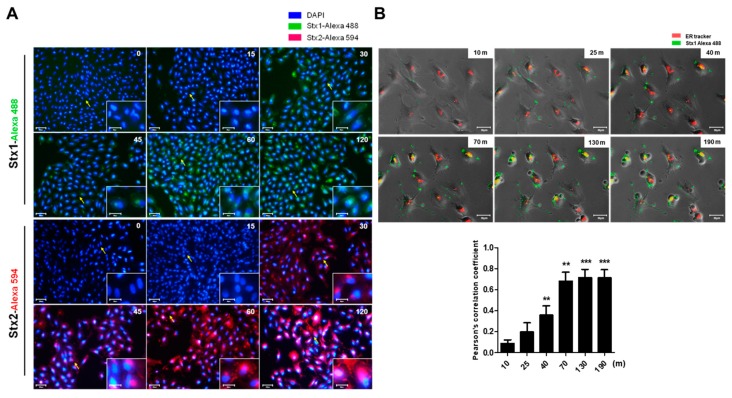
Detection of toxin intracellular trafficking to the ER in ARPE-19 cells treated with Stx1 or Stx2. (**A**) ARPE-19 cells were seeded in 12-well plates at a total cell density of approximately 1.0 × 10^5^ cells/well. Cells were incubated with Alexa Fluor 488-conjugated Stx1 (100 ng/mL) or Alexa Fluor 594-conjugated Stx2 (10 ng/mL) for the time points indicated in the right upper corner of each panel. After washing, cells were fixed and DAPI reaction solution added to stain nuclei. Representative DAPI-positive cells visualized by fluorescence microscopy are shown. In each panel, the arrow depicts cells shown in higher magnification in the insert. Scale bar: 80 μm, insert: 30 μm; (**B**) To detect Stx trafficking to the ER, 1.0 × 10^5^ ARPE-19 cells/well were seeded in 12-well plates. Subsequently, cells were stimulated with complete growth media containing Alexa Fluor 488-conjugated Stx1 (100 ng/mL) with 50 nM ER-tracker (red) live-cell staining dyes for detection of ER. After washing, cells were captured in time-lapse live imaging at the time points indicated in the right upper corner of each panel. Yellow fluorescence indicates co-localization of Stx1 with the ER-specific marker. The scale bars represent 30 μm. The bar graphs show the mean ± SEM of the Pearson’s correlation coefficient assessment of the co-localization of Stx1-Alexa Flour 488/ER tracker (lower panel). More than 30 cells were counted per condition (*n* = 3). Asterisks indicate significant differences between treated time at 10 min vs. indicated time points; ** = *p* < 0.01; *** = *p* < 0.001.

Previous studies have revealed that Stxs are internalized via Gb3 receptors to the *trans*-Golgi network and lumen of the endoplasmic reticulum (ER), a process is known as retrograde transport [6,33]. To visualize toxin internalization in ARPE-19 cells, we used immunofluorescence microscopy to examine the intracellular trafficking of Alexa Fluor 488-conjugated Stx1 or Alexa Fluor 594-conjugated Stx2 with 4′,6-diamidino-2-phenylindole (DAPI) co-staining. In ARPE-19 cells, we observed that Alexa Fluor 488-conjugated Stx1 and Alexa Fluor 594-conjugated Stx2 fluorescence were significantly increased in ARPE-19 cells over 30 min (Figure 3A). The fluorescence signal was confirmed to coalesce around the nucleus. In the live cell imaging studies, we observed that the perinuclear localization of Alexa Fluor 488-conjugated Stx1 co-localized with an ER specific [6] fluorescent marker, producing yellow fluorescence in merged images (Figure 3B, Movie S1). These experiments indicate that Stxs undergo retrograde intracellular trafficking to the ER in ARPE-19 cells.

### 2.4. Stx1 and Stx2 Activate Stress-Associated MAPKs in ARPE-19 Cells

**Figure 4:**
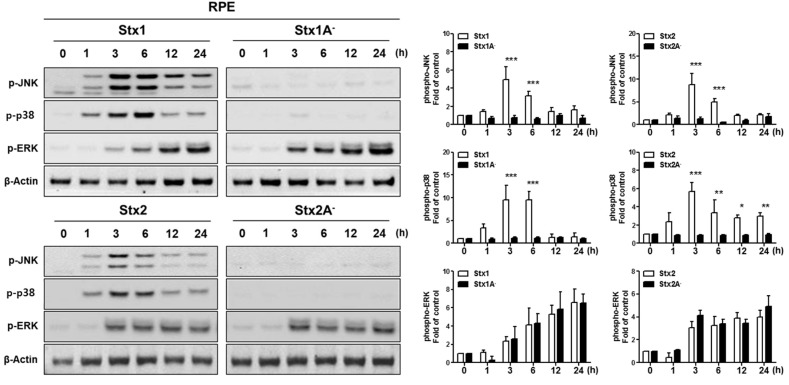
Stx1 and Stx2 activation of stress-associated MAPKs in RPE cells. ARPE-19 cells were stimulated with Stx1 (100 ng/mL), Stx1A^−^ (100 ng/mL), Stx2 (10 ng/mL) or Stx2A^−^ (10 ng/mL). After washing, cell lysates were collected at the indicated time points. Phosphorylation of p38MAPK (p-p38), Jun *N*-terminal kinase (p-JNK), and extracellular signal-regulated kinase (p-ERK) were examined by Western blotting. β-Actin was used as a control for equal protein loading. The results are from one representative experiment of three independent experiments. The data supports the necessity of Stx A_1_-fragment retro-translocation and action on the ribosomal peptidyl transferase center for activation of p38 and JNK, but not ERK. The bar graphs show the mean ± SEM of fold changes in band densities normalized to β-Actin in comparison to untreated control cell values (right panel). Asterisks indicate significant differences between Stx1 vs. Stx1A^−^ or Stx 2 vs. Stx2A^−^; * = *p* < 0.05; ** = *p* < 0.01; *** = *p* < 0.001.

In numerous studies, MAPK signaling has been shown to be involved in eliciting responses to various stresses [34]. In addition, the MAPK signaling pathways activated by Stxs trigger the ribotoxic stress response in various cell types [35,36]. We investigated whether Stxs activate MAPK signaling in ARPE-19 cells. Stxs induced the phosphorylation of p38, JNK and Extracellular signal–regulated kinase (ERK) after 1–3 h (Figure 4). Stxs with mutations in the enzymatic A subunits did not induce phosphorylation of p38 or JNK. In contrast, enzymatically inactive Stxs activated ERK phosphorylation. These results show that the internalization and intracellular trafficking of Stxs induce MAPK signaling, and Stx enzymatic activity is required for the activation of p38 and JNK, but not ERK. 

### 2.5. The ER Stress Response is Induced after Stx1 or Stx2 Exposure in ARPE-19 Cells

**Figure 5:**
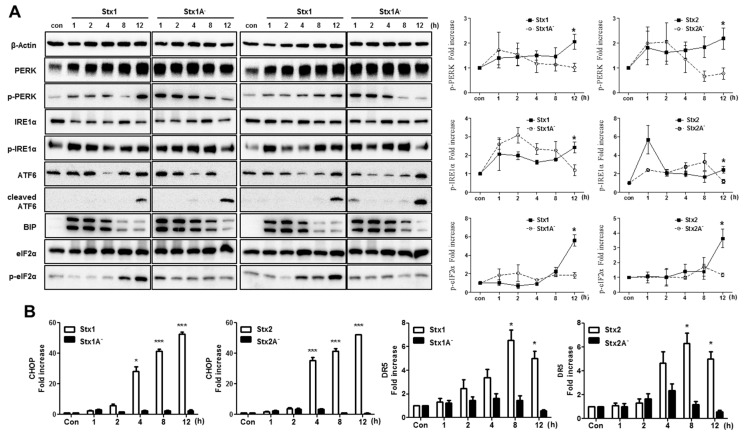
The ER stress response is induced in RPE cells after Stx1 or Stx2 exposure. (**A**) ARPE-19 cells (5.0 × 10^5^ cells/well) were stimulated with Stx1 (100 ng/mL), Stx1A^−^ (100 ng/mL), Stx2 (10 ng/mL) or Stx2A^−^ (10 ng/mL). At the indicated times (h), cells were lysed and the presence of activated ER stress sensors and their downstream targets in cellular lysates were determined by Western blotting. Untreated control cells (con) were used to determine baseline protein expression. β-Actin was used as a control for equal protein loading. The line graphs show the mean ± SEM of band densities normalized by the division of β-Actin band densities and compared to untreated control cell values (right panel). Statistical analyses of densitometric scans from at least three independent experiments are shown; (**B**) ARPE-19 cells were treated as outlined above and CHOP and DR5 mRNA expression were measured using RT-PCR and normalized using Glyceraldehyde 3-phosphate dehydrogenase (GAPDH). The data are expressed as mean and SEM increases in (**A** right panel) phospho-PERK (p-PERK), phospho-IRE1α (p-IRE1α), phospho-eIF2α (p-eIF2α), and (**B**) CHOP and DR5 mRNA levels compared to the levels in untreated control cells. Asterisks indicate significant differences between Stx1 and 2 vs. Stx1A^−^ and 2A^−^; * = *p* < 0.05; *** = *p* < 0.001.

Stxs that are trafficked to the ER have the capacity to induce the ER stress response through activation of the ER membrane-bound sensors protein kinase RNA-like endoplasmic reticulum kinase (PERK), inositol-requiring enzyme 1α (IRE1α), and activating transcription factor 6 (ATF6) in several types of cells [7]. If Stx-mediated ER stress is prolonged, it stimulates apoptotic signaling [37]. To assess the induction of ER stress in ARPE-19 cells, the cells were treated for 0 to 12 h with purified Stx1, Stx2, Stx1A^−^ or Stx2A^−^. We detected PERK activation that remained elevated up to 12 h after cells were treated with Stx1 or Stx2 (Figure 5A). However, Stx1A^−^ and Stx2A^−^ mediated the transient induction of PERK phosphorylation (p-PERK) that dramatically decreased 8 to 12 h after treatment. Consistent with these findings, we showed that Stx1 and Stx2 treatment induced prolonged activation (phosphorylation) of the PERK substrate eukaryotic translation initiation factor-2α (eIF-2α) (Figure 5A). We also observed IRE1α activation (p-IRE1α) that was increased up to 8 h after Stx1, Stx2, Stx1A^−^ or Stx2A^−^ treatment. However, when we treated ARPE-19 cells with holotoxins, activation of IRE1α was evident up to 12 h. Treatment of cells with Stx1 or Stx2 toxoids with A subunit mutations showed rapidly decreased IRE1α phosphorylation at 12 h (Figure 5A right panel). In contrast to these findings, we did not detect differences in ATF6 proteolysis between Stx1 or Stx2 and Stx1A^−^ or Stx2A^−^ (Figure 5A), suggesting that, following retrograde transport to the ER, individual toxin subunits may be sensed as proteins in an unfolded state in ARPE-19 cells. Immunoglobulin heavy chain binding protein (BiP) is a member of the Hsp70 ATP-dependent molecular chaperone family localized to the ER lumen. BiP is involved in both the translocation of newly synthesized polypeptides and retro-translocation of aberrantly folded proteins for degradation by the proteasome [38]. BiP appears to regulate the ER stress response by dissociation from PERK and ATF-6, and sequestration of the stress sensor IRE1α to prevent oligomerization and activation [39,40]. During ER stress, the dissociation of BiP from the stress sensors may activate transcription factors that markedly up-regulate BiP gene expression [41]. All of the Stx (Stx1, Stx2) or mutant toxin (Stx1A^−^ and Stx2A^−^) treatments induced higher expression levels of BiP compared to the results for control cells. Expression of the transcription factor C/EBP homologous protein (CHOP) is regulated by ER stress in response to unfolded proteins in ER [42]. Previous studies showed that Stx treatment of undifferentiated or macrophage-like THP-1 cells induced CHOP mRNA and protein expression [43]. Therefore, we determined whether CHOP mRNA expression is up-regulated in APRE-19 cells stimulated with Stxs (Figure 5B). CHOP mRNA expression dramatically increased 4 h after Stx1 or Stx2 treatment in ARPE-19 cells. Although treatment of APRE-19 cells with Stx1A^−^ or Stx2A^−^ mutant toxoids transiently activated the ER stress sensors PERK and IRE1α, the A subunit enzymatic mutants did not induce CHOP mRNA expression. ER-stress induced apoptosis is known to be involved in death receptor 5 (DR5) up-regulation by a CHOP dependent mechanism in some human cancer cells [44]. As shown in Figure 5B, increased levels of DR5 mRNA were detected beginning at 4 h and peaking at 8 h following stimulation with Stx1 or Stx2. However, Stx1A^−^ or Stx2A^−^ mutant toxoids did not induce DR5 mRNA expression. 

### 2.6. Stxs Activate Apoptotic Signaling Pathways in ARPE-19 Cells and Toxin Enzymatic Activity is Required for Apoptosis

**Figure 6:**
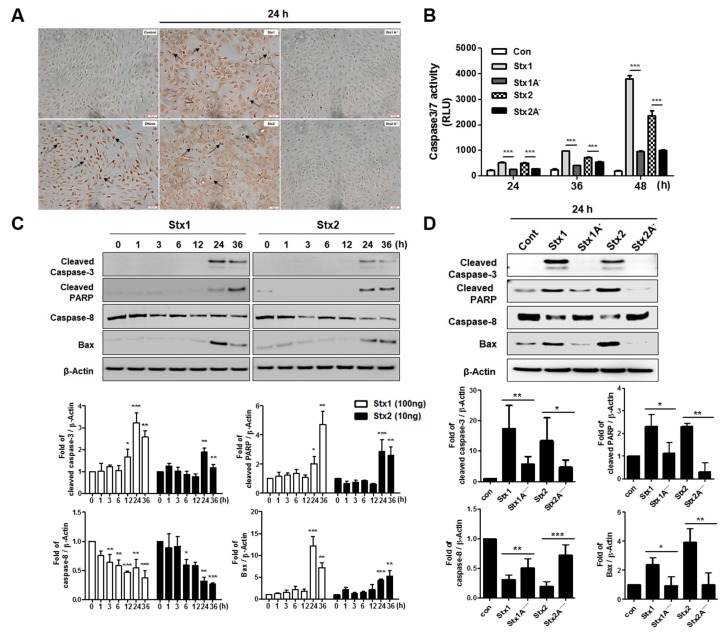
Stxs activate apoptotic signaling pathways in RPE cells. (**A**) The TUNEL assay was performed to detect apoptotic APRE-19 cells after treating with Stx1, Stx2, Stx1A^−^ or Stx2A^−^ for the indicated times. Cells staining dark brown are TUNEL positive and are indicated by arrows. Scale bars = 50 μm; (**B**) Analysis of caspase-3/7 activity. ARPE-19 cells were incubated with Stx1 (100 ng/mL), Stx1A^−^ (100 ng/mL), Stx2 (10 ng/mL) and Stx2A^−^ (10 ng/mL) for the indicated time points, and caspase 3/7 activity was measured using the Caspase-Glo 3/7 Assay; (**C**,**D**) ARPE-19 cells (1.0 × 10^5^ cells/well) were treated with Stx1 (100 ng/mL), Stx1A^−^ (100 ng/mL), Stx2 (10 ng/mL) and Stx2A^−^ (10 ng/mL) for the indicated time points. Protein samples were prepared and analyzed by Western blotting using anti-cleaved caspase-3, anti-caspase-8, anti-cleaved PARP, anti-Bax and anti-β-Actin antibodies. β-Actin was used as a control for equal protein loading. The results are from one representative experiment of three independent experiments. The bar graphs show the mean ± SEM of fold changes in band densities normalized by division of β-Actin band densities and compared to untreated control cell values (lower panels). Asterisks indicate significant differences between control cell values vs. Stxs-treated cells (panel **C**) or Stx 1 vs. Stx1A^−^ and Stx2 vs. Stx2A^−^ (panel **D**); * = *p* < 0.05; ** = *p* < 0.01; *** = *p* < 0.001.

Previous studies revealed that Stxs induce apoptotic cell death in human epithelial, endothelial and monocytic THP-1 cell lines [45]. We have shown that the internalization of Stxs in ARPE-19 cells induced stress-related MAPK signaling and ER stress protein activation. ER stress is known to induce apoptosis through p38 and JNK MAPK stress signaling [46]. ER stress-induced apoptosis can activate initiator caspase-8, a process that leads to the loss of mitochondrial membrane potential and activation of downstream effector caspase-3 [47]. Therefore, we explored whether Stx1 or Stx2 induces apoptosis in ARPE-19 cells. To analyze DNA fragmentation as an indicator of apoptotic cell death, we performed the terminal deoxynucleotidyltransferase-mediated 2′-Deoxyuridine, 5′-Triphosphate (dUTP)-biotin nick end labeling (TUNEL) assay. The TUNEL assays demonstrated increased TUNEL-positive APRE-19 cells 24 h after treatment with Stx1 or Stx2 compared to untreated control cells. Increased DNA fragmentation was not detected in Stx1A^−^ or Stx2A^−^ treated cells, suggesting that Stx enzymatic activity is required to induce apoptosis (Figure 6A). In order to confirm the occurrence of apoptosis, we also measured the activity of caspase-3/7. Caspase-3/7 activity in ARPE-19 cells increased progressively 12–48 h after treatment with Stx1 or Stx2. In contrast, caspase 3/7 activity in ARPE-19 cells treated with Stx1A^−^ or Stx2A^−^ slightly increased compared to control cells (Figure 6B). These results suggest that enzymatically active Stxs induce apoptosis in a caspase-dependent manner in ARPE-19 cells. As shown in Figure 6C, the uncleaved (inactive) form of caspase-8 significantly decreased beginning 3 h after incubation with Stx1 or Stx2. Since activated caspase-8 is known to cleave Bid, which in turn targets mitochondrial membrane perturbation and activates the pro-apoptotic protein Bax [48], we also confirmed that the expression of Bax was increased following the treatment of ARPE-19 cells with Stxs. Cleavage of caspase-3 and Poly (ADP-ribose) polymerase (PARP) was also observed by 24 h of treatment with Stxs. However, these reactions were not induced by treatment with Stx1A^−^ or Stx2A^−^ (Figure 6D). Interestingly, the levels of antiapoptotic protein Bcl-2 were decreased by Stx1 or Stx2 treatment in ARPE-19 cells while the levels of Bcl-2 were not altered by Stx1A^−^ or Stx2A^−^ (Figure S2). Because the Stxs activated the MAPK signaling in retinal pigment epithelial cell lines (see Figure 4), we performed inhibitor assays using SB203580, SP60125, U0126 to define the effects of MAPK signaling in Stx-induced apoptosis. ARPE-19 cells were treated with MAPK inhibitors and then incubated for 48 h with Stx1 or Stx2. Cell viability measurements indicated that p38 and JNK inhibitors reduced Stx1- and Stx2-induced cell death, but there was no significant difference in cell viability following ERK inhibition (Figure S3A). Also, we identified caspase-3/7 activation by Stx1 or Stx2 in MAPK inhibitor-treated ARPE-19 cells. Stx1/2-induced caspase-3/7 activity was reduced by p38 and JNK inhibitor treatments in ARPE-19 cells. Caspase-3/7 activity was less decreased when treated with ERK inhibitor in comparison to treatment with p38 and JNK inhibitors (Figure S3B). These results suggest that the MAPK signaling contributes to Stx-induced apoptosis. Taken together, these results suggest that Stxs, via a mechanism requiring enzymatic activity, induce apoptosis in a caspase-dependent manner in ARPE-19 cells.

### 2.7. Stxs Induce Loss of Mitochondrial-Membrane Potential

**Figure 7:**
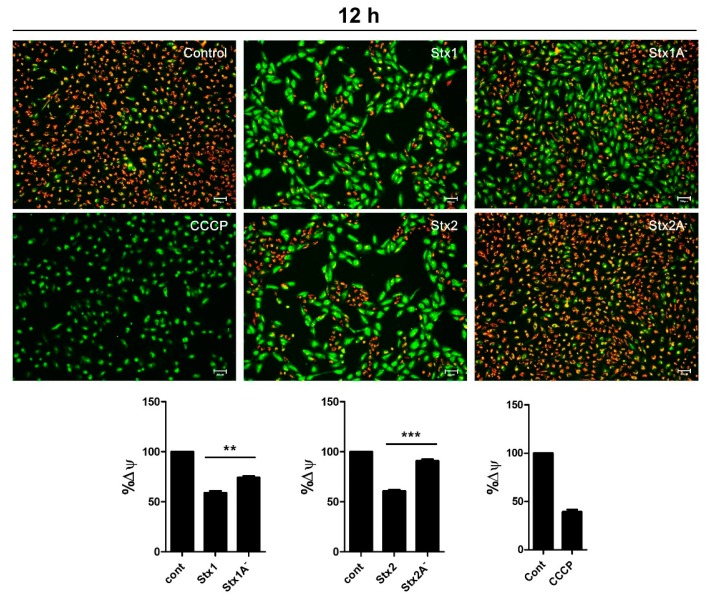
Stxs induced loss of mitochondrial membrane potential in RPE cells. Mitochondrial membrane potential in Stx-treated ARPE-19 cells was evaluated using JC-1 staining to monitor alterations of mitochondrial membrane potential. Red fluorescence indicates the accumulation of JC-1 aggregates in normal mitochondrial membranes. Green fluorescence indicates JC-1 monomers and membrane depolarization. ARPE-19 cells (1.0 × 10^5^ cells/well) were treated with Stx1 (100 ng/mL), Stx1A^−^ (100 ng/mL), Stx2 (10 ng/mL) and Stx2A^−^ (10 ng/mL) for 12 h. For the positive control, CCCP was added to 50 μM final concentration and cells were incubated at 37 °C for 5 min. The scale bars represent 200 μm. The quantitative analysis of JC-1 staining was measured by a microplate reader with 520 nm for emission of green fluorescence, and 590 nm for emission of red fluorescence (lower panel). The bar graphs show the mean ± SEM of percentages of cells with division of green fluorescence into red fluorescence normalized using untreated control cell values. Asterisks indicate significant differences between Stx1 vs. 1A^−^ or Stx2 vs. 2A^−^; ** = *p* < 0.01; *** = *p* < 0.001.

Mitochondria contain important regulators of apoptotic processes including caspases [49]. Loss of mitochondrial-membrane potential has been shown to contribute to apoptosis [50]. To assess the induction of mitochondrial depolarization in ARPE-19 cells, the cells were treated for 12 h with purified Stx1, Stx2, Stx1A^−^ or Stx2A^−^. Normal mitochondria with high Δψ_m_ accumulate red fluorescent JC-1 aggregates, whereas carbonyl cyanide m-chlorophenyl hydrazone (CCCP)-treated mitochondria with low Δψ_m_ display the green fluorescent monomeric form of JC-1. After Stx1 or Stx2 treatment, mitochondria showed an increase in monomeric green fluorescence and a decrease in JC-1 aggregated red fluorescence, indicating Δψ depolarization (Figure 7). These responses were not induced in Stx1A^−^ or Stx2A^−^ treated ARPE-19 cells (Figure 7). These findings demonstrate that Stx1- and Stx2-induced mitochondrial membrane depolarization is dependent on enzymatic activity.

## 3. Discussion

The role of Stxs in the diarrheal phase of disease and the myriad of systemic complications that may follow ingestion of toxin-producing bacteria remain to be fully characterized. In particular, the mechanisms by which the toxins mediate ocular disease are incompletely understood. A primary aim of this study was to explore retinal pigment epithelial cell responses to Stxs. In previous studies, we and others presented data to support the notion that Stxs are capable of inducing apoptosis (programmed cell death). Apoptosis is a well-characterized form of cell death that may follow activation of intracellular signaling pathways in response to a variety of cell stressors, including ER stress, in many different types of cells [15,51]. Besides the cell death signals triggered by Stxs through the ER stress response, the toxins as multifunctional proteins have also been shown to activate the ribotoxic stress response, the induction of autophagy and the elicitation of proinflammatory responses including the assembly of the NLRP3 inflammasome [52]. The capacity of Stxs to induce apoptotic signaling may play an essential role in pathogenesis including intestinal damage as well as extra intestinal complications such as HUS and the vascular damage implicated in the pathogenesis of CNS dysfunction [53]. For example, in HUS, extensive damage to glomerular endothelial cells and renal tubular epithelial cells was observed with pyknotic nuclei and sloughing of cells into the tubule lumina. A high coincidence between central nervous system disorders, which are associated with a higher rate of death, and cortical visual dysfunction has been reported in pediatric HUS patients [26], suggesting that ocular involvement may indicate a severe form of HUS. Previous ocular findings in HUS patients, including a 10-month-old infant with stools positive for STEC, consisted of retinal, choroidal and vitreal hemorrhages, as well as ischemic signs due to thrombotic microangiopathic lesions along with increased platelet consumption after initial vascular endothelial cell damage caused by Stxs [27,54,55]. Retinal pigment epithelial cells may be the targets of Stxs in disease progression that may follow the prodromal diarrheal disease. In the present study, we demonstrate that the ARPE-19 retinal epithelial cell line is sensitive to the cytotoxic action of Stx1 or Stx2, with estimated CD_50_s of 100 ng/mL and 10 ng/mL, respectively (Figure S1). Our results demonstrating cytotoxicity by utilizing light microscopy and MTS-based cell viability assays (Figure 1) suggested to us that the toxin receptor Gb3 should be expressed on the surface of ARPE-19 cells. In agreement with previous studies using various toxin-sensitive cell types [30], FACS analysis examining Gb3 expression on ARPE-19 cells showed high membrane expression of the Stx receptor in vitro (Figure 2). Purified fluorescently-labeled recombinant Stx1 and Stx2 proteins have been extensively employed to characterize toxin intracellular trafficking to different host cellular compartments [30,56]. As shown in Figure 3, Stx1 and Stx2 were internalized and routed to the ER in ARPE-19 cells, suggesting that rapid retrograde transport to the ER occurs with maximal fluorescence detected 40–190 min after the toxin exposure. This pattern of rapid intracellular toxin transport to the ER correlates with findings using other highly sensitive cell lines [57]. 

Smith et al. [29] associated the apoptotic cell death pathway with signaling through the ribotoxic stress response activated by exposure to Stx1 in the human epithelial cell line Hct8. Functionally active Stx1 holotoxin, but not an enzymatic activity deficient Stx1 mutant, triggered caspase-3 (ultimate executioner caspase) activation and nuclear fragmentation. Hct8 cells were partially protected from Stx1-induced apoptosis, with decreased caspase-3 activation and DNA fragmentation, when the cells were treated with a p38 MAPK inhibitor prior to exposure to the toxin [29]. The work of Iordanov et al. [14] suggested that ribosomal inactivating proteins that act on the ribosomal peptidyl transferase center (includes Stxs) activate stress-activated protein kinase cascades to initiate proinflammatory and proapoptotic signaling following the alteration of ribosomes. Accordingly, our findings summarized in Figure 4 specifically link Stx binding, internalization and retrograde transport to the ER with stress-activated protein kinase activation (ribotoxic stress response) in ARPE-19 cells. Interestingly, activation of p38 and JNK MAPKs requires toxin enzymatic activity, as toxoids with mutations that eliminate the ribosomal depurination reaction, fail to initiate signaling pathways leading to the phosphorylation of p38 and JNK. In contrast, ERK is activated in the absence of enzymatic activity, suggesting that intracellular routing to the ER or the ER membrane retrotranslocation process itself may be sufficient to trigger ERK signaling in APRE-19 cells. The comprehensive signaling cascades associating alterations in ribosomal function with MAPK activation in retinal epithelial cells exposed to Stxs remain to be fully characterized. There may be some cell-type specific responses among the multiple signaling pathways for the activation of the ribotoxic stress response. For example, using Hct8 and Vero cells, Jandhyala et al. demonstrated that DHP-2 (a pharmacological inhibitor of the upstream MAP3K ZAK) blocked Stx2-mediated activation of JNK and p38 MAPK, partially protected cells from apoptosis and partially reduced active caspase-3 levels without altering protein synthesis inhibition caused by the toxin [58].

In the ER, following synthesis by ribosomes, nascent polypeptides are folded correctly and the processed proteins transported to other locations in the cells or secreted. When overloading of the ER lumen with misfolded proteins occurs, it may lead to the induction of the ER stress response, also referred to as the unfolded protein response (UPR), to cope with the stress. Lee et al. used the toxin-sensitive monocytic THP-1 cell line to first report that, following binding to the membrane glycolipid Gb3 and transport to the ER, Stx1 was capable of inducing ER stress and activating the ER stress sensors PERK, IRE1 and ATF6 [11]. Based on these earlier findings, we initiated studies to characterize the ER stress response in RPE to define the ocular involvement of Stxs in visual impairment that may follow the ingestion of toxin-producing bacteria. We show here that Stx1 and Stx2 activate the ER stress sensors PERK, IRE1 and ATF-6 in ARPE-19 cells, along with enhanced expression of key downstream substrates of ER sensors BiP, CHOP (also called GADD153) and DR5 (TRAIL-R2) (Figure 5). In this regard, we speculate that the retrograde routing and enzymatic activity of functional Stxs may induce apoptosis via initiation of the ER stress response, leading to the rapid activation of the caspase3/7/8 cascade and the CHOP signaling pathways in retinal pigment epithelial cells. DR5 expression is regulated by CHOP [44], and we have previously demonstrated that Stx1 treatment of monocytic and macrophage-like THP-1 cells up-regulate the expression of CHOP and DR5 [11,37]. The precise role of TRAIL and DR5 in retinal pigment epithelial cells undergoing apoptosis induced by Stxs requires further scrutiny.

Apoptotic nuclei localized to tubular epithelial cells and glomerular endothelial cells were detected after TUNEL staining of renal cortical tissues in in vivo and in vitro studies [59]. Moreover, using renal biopsies from seven HUS patients, Te Loo et al. [60] subjected the tissue samples to dual staining with TUNEL and SC-35 (a dye to label for RNA synthesis and splicing factor to avoid non-specific TUNEL positive staining), and found that 80% of apoptotic cells were observed in tubules and 20% in glomeruli. The TUNEL analyses presented here suggest that Stx1- or Stx2-induced cell death is mediated by apoptosis, as evidenced by a marked increase of TUNEL^+^ cells following the intoxication of ARPE-19 cells. The induction of apoptosis in Stx-treated ARPE-19 cells was further characterized by caspase-3/7 activity measurement. Based on our observations, the enzymatic deficient variants (Stx1A^−^ and Stx2A^−^) could not activate either caspase-3 or caspase-8 processing whereas their active counterparts did activate these caspases. (Figure 6A,B). Rapid cleavage of pro-caspase-3 or pro-caspase-8, beginning 4–8 h after intoxication, was reported to occur coincidentally with the decreased expression of anti-apoptotic regulator Bcl-2 and the increased expression of pro-apoptotic factor Bax in Stx1-treated THP-1 cells [11,37]. We reasoned that if the intrinsic pathway was important in Stx-induced retinal pigment epithelial cell apoptosis, then ARPE-19 cells should have an increased loss of mitochondrial membrane potential (Δψ_m_), and we observed that this indeed was the case. Δψ_m_ was significantly disrupted when ARPE-19 cells were incubated with Stx1 or Stx2 holotoxin. However Δψ_m_ was not affected when the cells were exposed to Stx1A^−^ and Stx2A^−^ (Figure 7), supporting a requirement for toxin enzymatic activity to induce apoptosis in ARPE-19 cells. Similarly, we and other workers have previously shown that several toxin-sensitive cell types share functional apoptotic signaling mechanisms triggering major caspase activation and mitochondrial membrane depolarization after exposure to Stx1 or Stx2 [43,61,62]. 

**Figure 8:**
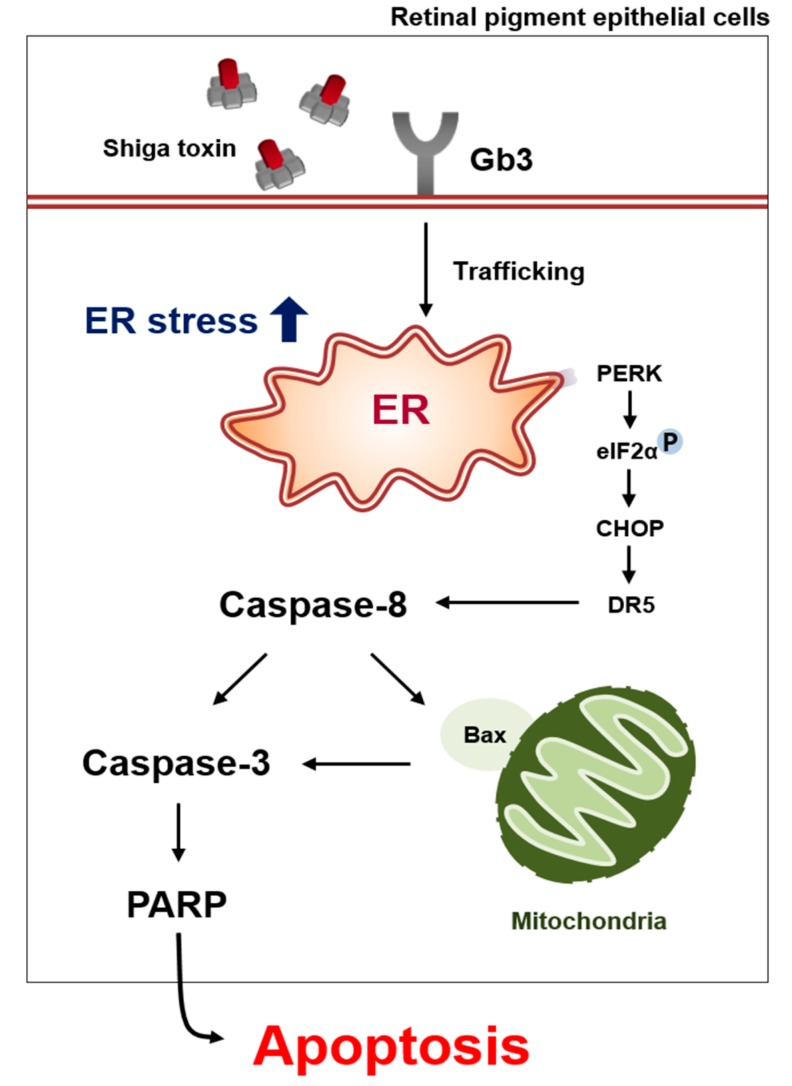
Proposed model of RPE apoptosis triggered by Stxs.

In conclusion, our data support the model of Stx1 or Stx2-induced apoptosis and ER stress pathways in human retinal pigment epithelial cells (Figure 8), suggesting the ocular involvement of Stxs in the pathogenesis of disease leading to visual impairment or blindness. Although there has been reported a high coincidence between neurological and visual system abnormalities, further experimentation will be required to clarify the pathophysiological role of Stxs in causing ocular disease. Funduscopy capable of observing the retinal microvasculature revealed retinal hemorrhages, attenuation of retinal venules, and extensive retinal detachment in the eyes of a patient infected with Shiga toxin-producing *Escherichia coli* O104 [9]. These clinical findings suggest that Stxs may be transmitted to the retinal epithelium via damaged ocular blood vessels. Alternatively, Stxs may disrupt the blood–brain barrier (BBB) due to thrombotic microangiopathic lesions formed after initial vascular endothelial injury. Landoni et al. [63] observed that Stx1 affected the permeability of the brain endothelium to influence the BBB once the toxin reached the brain parenchyma, contributing to the development of neuropathological symptoms observed in HUS. The defined location of Gb3 in the mammalian CNS, and the role of apoptosis in neuropathogenesis are controversial [41,64,65,66,67]. Unlike Stxs-mediated renal apoptosis, programmed neuronal cell death induced by Stxs has not been extensively characterized, although rabbit neurons appear to be susceptible to Stx2-induced apoptosis via focal proinflammatory response [68]. The identification of intermediate signaling molecules in Stx-induced retinal apoptosis may provide new insights into discovering therapeutic targets to disrupt the intoxicated cell death, thereby alleviating the retinal injury caused by the toxin. However, in human retinal pigment epithelium, many mechanistic aspects of the apoptotic signaling pathways initiated by Stxs remain to be explored. Moreover, further assessments to clarify the apoptosis signaling mechanisms activated by Stxs in in vivo retinal systems are needed.

## 4. Materials and Methods

### 4.1. Antibodies and Reagents

Rabbit monoclonal antibodies specific for human phospho-p38, phospho-p42/44(ERK), phospho-SAPK/JNK, cleaved caspase-3, cleaved caspase-8, cleaved caspase-9, cleaved PARP, Bax, Bcl-2, BiP, PERK, IRE1α, eIF-2α, phospho-eIF2α and mouse monoclonal anti-β-actin antibodies were purchased from Cell Signaling Technology (Danvers, MA, USA). Rabbit monoclonal antibodies specific for human ATF6 were purchased from Santa Cruz Biotechnology (Santa Cruz, CA, USA). Rabbit monoclonal antibodies specific for human phospho-IRE1α and phospho-PERK were purchased from Abcam (Cambridge, MA, USA). For mitochondrial membrane potential measurement, the Mitochondrial Membrane Potential Assay kit (Cell Signaling Technology, Danvers, MA, USA) using the JC-1 fluorescence was obtained to detect alterations in transmembrane potential.

### 4.2. Toxins

Stx1 was prepared as previously described [16]. Briefly, Stx1 was purified from cell lysates prepared from *E. coli* DH5α (pCKS112), a recombinant strain containing a plasmid encoding the *stx1* operon, by sequential ion exchange and immunoaffinity chromatography. The purity of toxin preparations was assessed by sodium dodecyl sulfate-polyacylamide gel electrophoresis (SDS-PAGE) with silver staining and western blotting analysis. Toxin preparations were passed through ActiClean Etox columns (Sterogene Bioseparations, Carlsbad, CA, USA) to remove trace endotoxin contaminants and were determined to contain <0.1 ng of endotoxin per mL as determined by the *Limulus* amoebocyte lysate assay (Associates of Cape Cod, East Falmouth, MA, USA). Purified Stx1 holotoxin containing a double mutation (E167Q and R170L) in the A subunit (Stx1A^−^) which dramatically reduces enzymatic activity was a kind gift from Dr. Shinji Yamasaki, Osaka Prefecture University, Osaka, Japan [69]. Recombinant purified Stx2, Stx2A¯ toxoid (Y77S/E167Q/R170L) were obtained from the NIAID, NIH Biodefense and Emerging Infections Research Repository (BEI Resources, Manassas, VA, USA).

### 4.3. Cytotoxicity Assay

ARPE-19 cells (5.0 × 10^4^ cells/well) were seeded in 96-well microtiter plates prior to treatment with Stx1 (100 ng/mL), Stx2 (10 ng/mL), Stx1A^−^ (100 ng/mL) or Stx2A^−^ (10 ng/mL). Cytotoxicity was determined by colorimetric assay [70] using the tetrazolium compound [3-(4,5-dimethylthiazol-2-yl)-5-(3-carboxymethoxyphenyl)-2-(4-sulfophenyl)-2*H*-tetrazolium] inert salt; (MTS, Promega, Madison, WI, USA). MTS (50 µL/5000 cells) were added to each well and incubation continued for 2 h at 37 °C in 5% CO_2_. Optical density was recorded with an automated microtiter plate reader at an absorbance of 490 nm (Microplate Reader with SoftMax; Molecular Devices, Sunnyvale, CA, USA). The percentage of cell death was determined using the following equation: percentage of cell death = [(average OD_490_ of treated cells − average OD_490_ of control cells) ÷ average OD_490_ of control cells] × 100. A background absorbance at 630 nm measured with untreated cells was subtracted from each sample reading. The reference wavelength 630 nm was used to subtract the background contributed by excess cell debris and other nonspecific absorbance.

### 4.4. Flow Cytometric Analysis of Gb3 (CD77) Membrane Expression 

ARPE-19 human retinal pigment epithelial cells were seeded at approximately 2.0 × 10^5^ cells/well into 12-well plates and cultured in Dulbecco's Modified Eagle Medium/Nutrient Mixture F-12 (DMEM/F12) media containing 10% FBS and supplemented with 5.0 μg/mL streptomycin and 5 U/mL penicillin. Cells were maintained at 37 °C in a humidified 5% CO_2_ atmosphere. After 24 h, ARPE-19 cells were mock-treated (basal level) or treated with Stx1 (100 ng/mL) or Stx2 (10 ng/mL) for 30 min or 1 h. After washing with cold PBS three times, ARPE-19 cells were detached with trypsin-EDTA and collected by centrifugation at 780× *g* for 5 min. Then cells were stained with Alexa Fluor 647-conjugated anti-human Gb3/CD77 monoclonal antibody (BD Biosciences, San Jose, CA, USA) or Alexa Fluor 647-conjugated mouse IgM (isotype control) at 4 °C for 30 min in the dark and analyzed by flow cytometry using a BD FACSCanto^TM^ ІІ cytometer (BD Bioscience, San Jose, CA, USA).

### 4.5. Intracellular Trafficking Assay

Intracellular trafficking of Stxs into RPE cells was determined using purified Stx1 conjugated to fluorescent tags (Alexa488 and Alexa594, respectively). Purified Stx1 or Stx2 were labeled with Alexa Fluor-488 or -594 dyes (Molecular Probes, Inc., Invitrogen, Eugene, OR, USA) as described in the manufacturer’s protocol. Briefly, ARPE-19 cells (1.0 × 10^5^ cells/well) were seeded overnight into 12-well plates. The cells were washed twice with PBS and incubated in complete DMEM/F12 growth media supplemented with 0.5% FBS before further staining for 30 min at 37 °C in the presence of 5% CO_2_. Subsequently, cells were treated with Alexa Fluor 488-conjugated Stx1 (100 ng/mL) or Alexa Fluor 594-conjugated Stx2 (10 ng/mL) for the indicated time points. Cells were washed in PBS and fixed in 4% paraformaldehyde in PBS for 10 min at room temperature and then cell nuclei were labeled by DAPI staining. Fluorescently conjugated Stx1 or Stx2 was observed by fluorescence inverted microscopy. To detect Stx trafficking to the ER, ARPE-19 cells were treated with Alexa Fluor 488-conjugated Stx1 (100 ng/mL) with an endoplasmic reticulum-specific dye (50 nM ER-Tracker Live Cell Staining dyes; Molecular Probes, Inc., Eugene, OR, USA). Using time-lapse live-cell imaging microscopy (EVOS^®^ FL Auto Cell imaging System, Thermo Fisher Scientific, Waltham, MA, USA), ARPE-19 cells were extensively imaged over the next 190 min. Binding competition between the anti-Gb3 antibody and the Stxs at the cell surface was observed, which prevents the use of the antibody to observe the intracellular colocalization of the receptor Gb3 and the toxins. The images shown are representative of at least two independent experiments. All data within each experiment were collected at identical settings.

### 4.6. Western Blotting

The cells were stimulated with Stxs, harvested, and lysed in a CHAPS buffer supplemented with protease and phosphatase inhibitors (GenDEPOT, Barker, TX, USA). Equal amounts of proteins (70–100 µg/lane) were separated by 8% or 12% Tris-glycine SDS–PAGE and transferred to nitrocellulose membranes. Membranes were blocked with 5% non-fat milk prepared with TBST (20 mM Tris (pH 7.6), 137 mM NaCl, 0.1% Tween 20). Membranes were incubated with primary antibodies at 4 °C for 24 h. After washing, the membranes were incubated with horseradish peroxidase-labeled secondary antibodies for 2 h at room temperature. Bands were visualized using the Western Lightning Chemiluminescence System (Atto Co., Tokyo, Japan). Data were obtained from at least three independent experiments. The integrated density was measured using the ImageJ software (NIH, Bethesda, Rockville, ML, USA).

### 4.7. Real-Time Quantitative RT-PCR

ARPE-19 human retinal pigment epithelial cells were seeded at approximately 2.0 × 10^5^ cells/well into 12-well plates and treated with Stx1 (100 ng/mL), Stx1A^−^ (100 ng/mL), Stx2 (10 ng/mL) or Stx2A^−^ (10 ng/mL). At the indicated times, total RNA was extracted using a RNeasy mini kit (Qiagen, Hilden, Germany) and cDNA was synthesized using ReverTra Ace-α-^®^ reverse transcriptase (Toyobo Life Sciences, Osaka, Japan). Real-time PCR was performed using the ViiA7 real-time PCR system (Life Technologies Corporation, Carlsbad, CA, USA) and Thunderbird SYBR qPCR Mix (Toyobo Life Sciences). The following primers were employed in these reactions: Human CHOP mRNA 5′-TAGGGGACATGTGTGAGCATGA-3′ (sense) and 5′-TCACGGCAAAGAGA TCGGAGA-3′ (anti-sense); Human DR5 mRNA 5′-AAGACCCTTGTGCTCGTTGT-3′ (sense) and 5′-GGAGCTAGGTCTTGTTGGGT-3′ (anti-sense); Human GAPDH mRNA 5′-GCACCGTC AAGGCTGAGAAC-3′ (sense) and 5′-TGGTGAAGACGCCAGTGGA-3′ (anti-sense). The relative mRNA levels for all genes were normalized to the levels of GAPDH.

### 4.8. TUNEL Assay

For terminal deoxynucleotidyltransferase-mediated dUTP-biotin nick end labeling (TUNEL) analysis, ARPE-19 cells were grown on glass coverslips. Cells were treated with Stx1, Stx1A^−^, Stx2 and Stx2A^−^ for 12 and 24 h. After fixing with 4% paraformaldehyde for 10 min, the coverslips were washed twice with 1× PBS for 5 min, and permeabilized with 0.2% Triton X-100 in PBS for 5 min at room temperature and then analyzed using a DeadEnd Colorimetric TUNEL Analysis Kit (Promega, Madison, WI, USA). 

### 4.9. Caspase 3/7 Activity

Apoptosis induction of ARPE-19 cells was measured via caspase activity. Cells were seeded at approximately 5.0 × 10^5^ cells/well in 6-well plates, cultured overnight and then treated with Stx1 (100 ng/mL), Stx1A^−^ (100 ng/mL), Stx2 (10 ng/mL), or Stx2A^−^ (10 ng/mL) in DMEM/F12 containing 0.5% FBS for the indicated times. The cells were washed three times with PBS and lysed with Lysis buffer containing protease inhibitors. Caspase activity was assessed using a luminescent assay (Caspase-Glo 3/7 Assay, Promega Corp., Madison, WI, USA) according to the manufacturer’s directions.

### 4.10. Mitochondrial Membrane Potential Assay

The lipophilic, cationic dye JC-1 (5,5′,6,6′-tetrachloro-1,1′,3,3′-tetraethylbenzimidazolcarbocyanine iodide) [71] fluorescence associated with mitochondrial membranes was detected using immunofluorescence microscopy. Red fluorescence indicated healthy cells and green fluorescence indicated cells with mitochondrial membrane dysfunction. ARPE-19 cells were seeded and treated with Stxs for 12 h. After treatment, cells were incubated with JC-1 to produce a final concentration of 2.0 μM for 30 min at 37 °C in the presence of 5% CO_2_. The plates were washed once with warm PBS. The quantitative analysis of the JC-1 staining was measured by a microplate reader with 514 nm for excitation and 529 nm for emission of green (monomer form) fluorescence, and 585 nm for excitation and 590 nm for emission of red (aggregate form) fluorescence. The percentage change of the mitochondrial transmembrane potential was determined using the following equation: percentage of Δψ_m_ = (average OD_514–529_ of treated cells ÷ average OD_585–590_ of control cells) × 100.

### 4.11. Quantitative Analysis

Quantitative analysis of the images was carried out using ImageJ. Pearson’s correlation coefficients of multiple sets of images were quantified using the ‘Colocalization’ tool in the ImageJ. The values were between 0 and 1; a value of 1 meant complete co-localization, while a value of 0 meant no co-localization.

### 4.12. Statistical Analysis

The differences among the mean values of the different groups were assessed, and all data are expressed as mean ± SEM. All of the statistical calculations were performed by one-way ANOVA followed by the Tukey post test using GraphPad Prism version 5.00. (GraphPad Software, Inc., La Jolla, CA, USA). Values of * *p* < 0.05 were considered statistically significant. (* = *p* < 0.05; ** = *p* < 0.01; *** = *p* < 0.001).

## Supplementary Materials

The following are available online at www.mdpi.com/2072-6651/9/10/319/s1, Figure S1: Dose- and time-dependent cytotoxic effects of Shiga toxin in ARPE-19 cells, Movie S1: Live imaging movie of intracellular trafficking to the ER in RPE cells treated with Stx1, Figure S2: In ARPE-19 cells, protein levels of pro-survival factor Bcl-2 were significantly decreased by Stx1 or Stx2, Figure S3: Stx1 and Stx2-induced cell death and caspase3/7 activity were dependent on MAPK signaling in ARPE-19 cells.
