# Supplementary material for: Shiga Toxins Induce Apoptosis and ER Stress in Human Retinal Pigment Epithelial Cells
Source: Toxins (Basel). 2017 Oct 13;9(10):319. doi: 10.3390/toxins9100319 (PMC5666366; doi:10.3390/toxins9100319)
Supplement: Supplementary file 1 [file toxins-09-00319-s001.zip › toxins-202653-supplementary/Supplementary_information_toxins 202653_round2.pdf]

Supplementary Materials: Shiga Toxins Induce Apoptosis and ER Stress in Human Retinal Pigment Epithelial Cells

Jun-Young Park, Yu-Jin Jeong, Sung-Kyun Park, Sung-Jin Yoon, Dae Gwin Jeong, Su Wol Chung, Byung Joo Lee, Jeong Hun Kim, Vernon L. Tesh, Moo-Seung Lee and Young-Jun Park

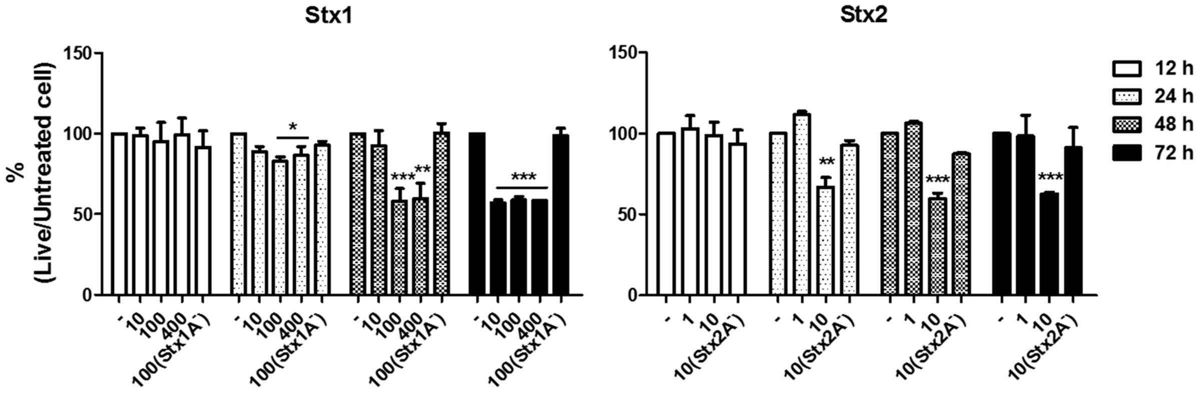

Figure S1. Dose- and time- dependent cytotoxic effects of Shiga toxin in ARPE-19 cells. ARPE-19 cells were seeded in 96-well plates the total cell density of 1.0 × 10^4 cells/well. Cells were incu bated with Stx1, Stx1A¯¯, Stx2 and Stx2A¯¯ for indicated dose. Supernatants were harvested fo r the cell incubated with or without Stxs for 12, 24, 36, and 48 h. Cell viability was measured by colorimetric assay using the dye MTS. Data are expressed as % viability compared to untre ated control cells at each time point. Results shown are means ± SEM from 2 independent exp eriments using triplicate samples. Statistical comparison was calculated using Students *t*-test (* = $p < 0.05$, ** = $p < 0.01$, and *** = $p < 0.001$).

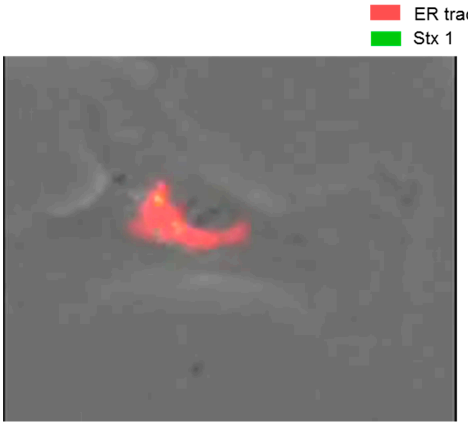

Movie S1. Live imaging movie of intracellular trafficking to the ER in RPE cells treated with S tx1. To detection of Stx1 trafficking of ER, 1.0 × 10^5 cells/well of ARPE-19 cells were seeded in 12-well plates. Subsequently, cells were stimulated with complete growth media containing Al exa Flour 488-conjugation Stx1 (100ng/ml) with 50 nM ER-Tracker (red) live-cell staining dyes for detection of ER for 10 min at 37℃ in a humidified 5% CO_2 atmosphere.. After washing, cells were captured in time-lapse live imaging during every 15 min for 3h. Time-lapse movies of ARPE-19 cells were acquired using the Evos® FL Auto Imaging System.

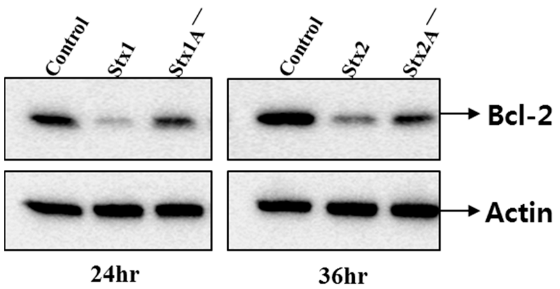

Figure S2. In ARPE-19 cells, protein levels of pro-survival factor Bcl-2 were significantly decreased by Stx1 or Stx2. The cells were stimulated with Stx1 (100 ng/ml), Stx1A^{-} (100 ng/ml), Stx2 (10 ng/ml) or Stx2A^{-} (10 ng/ml) for 24 or 36 h. Cell lysates were prepared for Western blotting performed using antibody against Bcl-2. Blots were stripped and reprobed with antibody against Actin for equal protein loading. Control, lysates prepared from control cell maintained for 24 or 36 h without toxin.

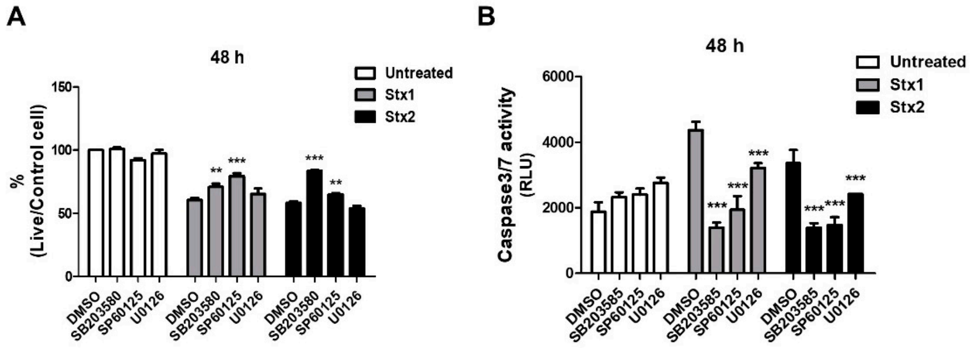

Figure S3. Stx1 and Stx2-induced cell death and caspase3/7 activity were dependent on MAPK signaling in ARPE-19 cells. ARPE-19 cells were seeded in 96-well plates at a total cell density of approximately 1.0×10^4 cells/well. Cells were incubated with 10μM of SB203580 (p38 inhibitor), SP60125 (JNK inhibitor) and U0126 (ERK inhibitor) for 2 h. And then, cells were treated Stx1 (100 ng/ml) and Stx2 (10 ng/ml) for 48 h. (A) Cell viability was measured by colorimetric assay using the dye MTS. Data are expressed as % Live/Control cell compared to untreated control cells. (**B**) Caspase 3/7 activity was investigated by using Caspase-Glo 3/7 Assay. Results shown are means ± SEM from two independent experiments using triplicate samples. Asterisks denote statistical significance, DMSO *vs.* SB203580, SP60125 and U0126 treatments (** = $p < 0.01$; *** = $p < 0.001$).
